# Cognitive and Behavioral Patterns across Psychiatric Conditions

**DOI:** 10.3390/brainsci11121560

**Published:** 2021-11-25

**Authors:** Roee Admon, Oded Klavir

**Affiliations:** 1 School of Psychological Sciences, University of Haifa, Haifa 3498838, Israel; 2 The Integrated Brain and Behavior Research Center (IBBRC), University of Haifa, Haifa 3498838, Israel

Psychiatric conditions represent a highly heterogeneous group of disorders associated with chronic distress and a sharp decline in quality of life. All psychiatric conditions involve various combinations of maladaptive cognitive and behavioral patterns that may appear at different shapes and intensities within a given condition. Notably however, many maladaptive cognitive and behavioral patterns are also present across different psychiatric conditions. This, in turn, presents substantial challenges for current diagnostic and therapeutic efforts, and may partly account for their limited efficacy. In recent years it has become increasingly clear that studying cognitive and behavioral patterns across psychiatric conditions, rather than studying a specific psychiatric condition per se, could substantially increase our understanding of the underlying mechanisms of mental disorders, and hence contribute to improved clinical care. In the current Special Issue, we included eight original research articles, two perspectives and one review paper, together highlighting novel findings and concepts within this broad field of research.

This scientific approach is nicely illustrated by studies that investigate specific cognitive patterns that are known risk factors for multiple psychiatric conditions. To this end, Schettino and colleagues focus on perseverative cognition, a form of cognition that is characterized by repetitive, intrusive and negative thoughts and has been linked to depression and anxiety. By combining laboratory-based reward tasks, computational modelling and ecological assessments in daily life, the authors demonstrate, for the first time, that perseverative cognition is also associated with abnormalities in the functionality of positive valence systems [1]. Another example stems from the study of Saporta and colleagues that focuses on loneliness. Loneliness is associated with adverse effects on physical and mental health that may lead to psychopathology. During the era of COVID-19, loneliness related to social isolation afflicted large portions of society. This enabled the authors to study and compare loneliness both as situational condition (as manifested by COVID-19) and as a chronic trait. Interestingly, opposing effects of situational vs. chronic loneliness emerged, such that situational loneliness led people to reconnect with their peers, while chronic loneliness pushed people away from each other [2]. Another vulnerability factor for multiple psychopathologies is impulsivity, investigated here in the study by Simon and colleagues. At first, the authors demonstrate that exposure to acute stress is associated with an increased tendency to act in an impulsive manner, consistent with previous findings. However, a more thorough examination that takes into account inter-individual variability in the impact of stress on impulsive behavior revealed quadratic relations between stress and impulsivity. Specifically, elevated physiological and psychological responses to acute stress were found to be associated with either increased or decreased choice impulsivity after stress [3]. Another highly relevant construct is drug consumption, particularly psychoactive drugs, as it may increase the likelihood of psychopathology and even suicidality. Martinotti and colleagues assessed suicidality prevalence among drug users that were admitted to a psychiatry ward in a hospital in Ibiza during the years 2015–2019. Interestingly, suicidality was present in 39% of the study cohort, yet suicidality rates were not related with previous or ongoing psychopathological symptoms, suggesting that impulsivity and loss of self-control may be determinants of suicidality irrespective of psychiatric background [4]. Finally, threat learning is an important cognitive construct in the development of normative fear and pathological anxiety. Threat learning can be achieved by either direct or vicarious (observational) learning. In their comprehensive review paper, Skversky-Blocq and colleagues highlight novel behavioral and neuroimaging literature on observational threat learning, focusing on dynamics in this learning pathway across human development. The authors conclude by discussing how observational threat learning may contribute to the development of normative fear and pathological anxiety [5]. 

Considering that many cognitive and behavioral patterns are well-conserved across species, and that there are homologies between the neural mechanisms of these functions, animal studies can substantially contribute to the identification of underlying biological substrates. An excellent example of this approach stems from the examination of the neural circuitry of action control that has been implicated as underlying compulsive behavior. In this Special Issue, Lousada and colleagues describe the results of structural characterization of myelin thickness and axon area in both associative and sensory-motor cortico-striatal circuits of the Sapap3 knockout (Sapap3-KO) mouse. This mouse model is currently the first-line model for compulsivity-related repetitive behaviors. Interestingly, the authors found that axon caliber, the main contributor to changes in conduction speed, is only specifically reduced in the associative striatum of the Sapap3-KO mouse [6]. This, in turn, implies that compulsive behavior may emerge from a connectivity deficit in the associative striatum rather than in the sensory-motor striatum. Bar Or and Klavir focus on another aspect of animal studies in this field, by looking at basic animal behavioral mechanisms. Using a novel learning paradigm, they were able to examine how different behaviors are affected by different behavioral control mechanisms, representing either goal-directed or goal-insensitive habitual control. The authors demonstrated that excessive practice of instrumental conditioning may form habitual patterns that cannot be amended, even when resulting in a noxious outcome, in contrast to consummatory or Pavlovian behaviors that maintain the ability to adapt [7]. 

A third line of research that is covered in this Special Issue relates to studies that were conducted directly in psychopathological populations. In the study by Preglej and colleagues, patients with mesial temporal lobe epilepsy (MTLE), with or without depression, were compared to healthy controls with respect to free recall and self-relevance ratings of emotionally-valenced words. By that, the authors were able to demonstrate that MTLE patients with depression endorse fewer positive words and more negative words as self-relevant, and self-evaluate their cognition as poorer, compared to MTLE patients without depression and healthy controls [8]. Levi and colleagues followed fluctuations in negative and positive affect, as well as in salivary cortisol, among depressed patients throughout psychotherapy. They also assessed the therapists’ cortisol level at each timepoint. Using this novel approach, the authors were able to demonstrate that increases in cortisol levels during a therapeutic session in patients were associated with an elevated negative affect during that session only when the therapist exhibited a decrease in cortisol levels. In contrast, increases in cortisol levels were associated with an elevated positive affect when the therapist showed an increase in cortisol [9]. These findings provide novel evidence for the importance of social context in the cortisol–affect relationship among depressed patients undergoing psychotherapy.

The two perspective papers that are included in this Special Issue also provide an overview of related topics. Horesh and colleagues point towards emotional contagion and symptom transmission in psychopathology, accounting for the impact of patients’ psychopathological status on others in their close environment. In their comparative theoretical analysis the authors provide strong evidence to support symptom transmission trans-diagnostically, across four distinct psychiatric disorders: posttraumatic stress disorder, major depression, obsessive compulsive disorder and psychosis. The authors further highlight the shared and differential mechanisms underlying these processes across the four disorders [10]. Finally, Djerassi and colleagues propose a novel neuro-cognitive model for autism that focuses on impaired social learning in infancy as a prominent contributor to autism development. The authors support their claims by reviewing novel evidence from neuroscience and developmental science that demonstrate how typical social development depends on two domain-general processes, motivation and multi-modal associations, processes that might be impaired among individuals with autism [11].

Taken together, the current Special Issue integrates novel evidence from animal studies and human studies among healthy and psychiatric populations. These studies explore different cognitive and behavioral patterns that are relevant in the prediction, maintenance, and recurrence of multiple psychiatric conditions. The studies in this issue further highlight several biological substrates of these patterns, at the neurobiological, physiological, endocrinological and genetic levels. Improved understanding of these mechanisms across psychiatric conditions may eventually lead to better utilization of scientific findings in clinical practice. This progress may emerge in the form of earlier and more accurate identification of individuals at risk for psychopathology, as well as in the form of efficacious, individually tailored treatment selection and personalized medicine in psychiatry.
